# USP1 Maintains the Survival of Liver Circulating Tumor Cells by Deubiquitinating and Stabilizing TBLR1

**DOI:** 10.3389/fonc.2020.554809

**Published:** 2020-09-25

**Authors:** Yuancheng Li, Yang Xu, Chao Gao, Yunfan Sun, Kaiqian Zhou, Pengxiang Wang, Jianweng Cheng, Wei Guo, Cao Ya, Jia Fan, Xinrong Yang

**Affiliations:** ^1^Institutes of Biomedical Sciences, Fudan University, Shanghai, China; ^2^Key Laboratory of Carcinogenesis and Cancer Invasion, Department of Liver Surgery, Ministry of Education, Zhongshan Hospital, Liver Cancer Institute, Fudan University, Shanghai, China; ^3^Department of Laboratory Medicine, Zhongshan Hospital, Fudan University, Shanghai, China; ^4^Key Laboratory of Carcinogenesis and Cancer Invasion, Ministry of Education, Cancer Research Institute, Central South University, Changsha, China

**Keywords:** circulating tumor cells, deubiquitination, hepatocellular carcinoma, USP1, Wnt pathway

## Abstract

The prognosis of hepatocellular carcinoma (HCC) is closely associated with the occurrence of distant metastases, which is likely due to circulating tumor cells (CTCs). However, the low number of CTCs is the main obstacle limiting research of the mechanism of CTC metastasis. Here, We evaluated the role of ubiquitin-specific protease 1 (USP1) in promoting CTC survival during blood-borne metastases. We observed that USP1 was frequently upregulated in CTCs and correlated with metastasis and a reduced overall survival rate of patients. Additionally, genetic knockout of *USP1* the survival rate of CTCs. Further analyses showed that USP1 mediates oncogenic activity by deubiquitinating and stabilizing transducin β-like 1 X-linked receptor 1 (TBLR1), which plays essential roles in regulating Wnt signaling. These results demonstrated that USP1 may act as an essential factor in promoting the survival of CTCs and suggest that inhibition of USP1 is a potential strategy for HCC treatment.

## Introduction

Hepatocellular carcinoma (HCC) is among the most prevalent malignancies worldwide accounting for >90% of human liver cancer cases. The morbidity and mortality rates of HCC has increased in recent decades (1). HCC has a high risk of metastasis, especially intrahepatic metastasis, and recurrence, which are the primary causes of death (2). Dissemination of circulating tumor cells (CTCs) is highly correlated with cancer metastasis and recurrence (3). Enumeration of CTCs is currently performed to monitor the anticancer treatment response and guide the prognosis of patients (4). Further investigation of the CTC survival mechanism may improve our understanding of metastasis and lead to new cancer therapies targeting CTCs. However, the number of CTCs is very low (1–10 single CTCs per 7.5 mL blood) and the lack of CTCs to analyze is the main obstacle to studies of the survival mechanisms of CTCs in blood-borne metastasis (5, 6).

Deubiquitination, a highly regulated process, is essential for maintaining cellular homeostasis via the regulation of numerous cellular functions, including protein levels, apoptosis, DNA repair, and cell motility (7–11). Ubiquitin-specific protease 1 (USP1), a sub-type of deubiquitinases, reportedly regulates DNA-repair processes by deubiquitinating proliferating cell nuclear antigen and Fanconi anemia group D2 and preserves cancer stem cells in osteosarcoma by stabilizing inhibitor of DNA binding (ID)1 and ID2 (12, 13). However, few studies have examined USP1-related function in HCC and/or the mechanism of CTC survival. We demonstrated that USP1 promotes the survival of liver CTCs in the bloodstream by regulating the ubiquitination of transducin β-like 1 X-linked receptor 1 (TBLR1), a critical regulator of the Wnt pathway, suggesting USP1 as a potential target for anticancer therapy.

## Materials and Methods

### Patients and Specimens

For immunohistochemistry (IHC) assay, from 2002 to 2008, 217 tissue specimens from patients with HCC were collected at the Zhongshan Hospital (Shanghai, China). For CTC analytical assay, blood (7.5 mL) was obtained from the peripheral veins of patients from 2017 to 2018. This study was approved by the Research Ethics Committee of the Zhongshan Hospital (B2017-159R), and the procedures were in accordance with the ethical guidelines outlined in the 1975 Declaration of Helsinki.

### Cell Culture and Construction of USP1-Knockout Cells

The PLC/PRF/5 human HCC cell line and 293T cell line were purchased from American Type Culture Collection (ATCC, Manassas, VA, United States) and the MHCC-97H cell line was obtained from the Liver Cancer Institute of the Zhongshan Hospital, Fudan University (Shanghai, China). MHCC-97H-GFP and PLC/PRF/5-GFP cell lines were constructed by lentiviral transfection, and MHCC-97H and PLC/PRF/5 *USP1*-knockout (KO) cell lines were constructed using CRISPR technology as follows: MHCC-97H and PLC/PRF/5, wild-type cell lines, were transfected with a *USP1*-targeting KO plasmid. Following digestion, single cells were seeded into a well, and after reaching confluence, sequencing was performed to confirm the construction of the KO cell lines (14).

### RNA Extraction and qRT-PCR

Total RNA was extracted using TRIzol reagent (Ambion, Austin, TX, United States). cDNA of the target gene was reverse-transcribed from total RNA (1 μg) using the Transcriptor reverse transcriptase kit (RR036A; TaKaRa, Shiga, Japan). Single CTCs from each patient were transferred to individual PCR tubes containing lysis buffer by micromanipulation. Single CTCs from each patient were transferred individually to single PCR tubes containing lysate buffer. Single cell RNA was extracted from each CTC and reverse-transcribed by Single Cell-to-CT qRT-PCR kit (Invitrogen, Carlsbad, CA, United States) following the manufacturer’s protocols. SYBR Green (Bio-Rad Laboratories, Hercules, CA, United States) and ABI Prism 7500 real-time PCR (Bio-Rad) were used for single-step qRT-PCR. Gene expression was calculated relative to that of *β-actin* expression using the 2^–ΔΔ*Ct*^ method.

### Tissue Microarrays, Immunohistochemistry, and Evaluation

An immunohistochemistry assay was performed. Briefly, serial-sectioning of tissue samples was performed after fixation in paraffin using 4% paraformaldehyde, microwave antigen retrieval was performed and the samples were incubated overnight with primary antibody followed by 1 h incubation with secondary antibodies. All tissues were counterstained with hematoxylin. The antibodies used in IHC assay included anti-USP1 (1:300; Proteintech, Rosemont, IL, United States), anti-TBLR1 (1:300; Proteintech).

### Circulating Tumor Cells Capture and Fluorescence-Activated Cell-Sorting

Circulating tumor cells were enriched from 7.5 ml blood samples by Ficoll solution (Sigma-Aldrich, St. Louis, MO, United States), incubated with fluorescent antibodies include anti-cytokeratin 19 (1:300; Cell Signaling Technology, Danvers, MA, United States), anti-EpCAM (1:300; Cell Signaling Technology), anti-CD45 (1:300; Cell Signaling Technology), and captured by flow cytometry (BD Biosciences, San Diego, CA, United States). The criteria for identifying the captured cells as the CTCs were: EpCAM positive; pan-cytokeratin-19 positive; CD45 negative; the presence of a nucleus, stained using 4’,6-diamidino-2-phenylindole (DAPI) (15). GFP cells sorted from mouse blood after injection or cultured GFP cells harvested from plates, were evaluated by Annexin V-Allophycocyanin (APC)/7-Aminoactinomycin D (7-AAD) kit (BD Biosciences Pharmingen, San Diego, CA, United States) following the manufacturer’s instructions and FlowJo software (TreeStar, Ashland, OR, United States) was used for data analysis.

### Western Blot Analysis

Cells were lysed using RIPA buffer (Beyotime, Nantong, China) containing 1 mM phenylmethylsulfonyl fluoride (Beyotime). Protein was loaded and separated by 8% or 12% SDS-PAGE gels, transferred to polyvinylidene fluoride membranes (Millipore, Darmstadt, Germany), and blocked with bovine serum albumin (5%; Sangon Biotech, Shanghai, China). The primary antibodies included anti-USP1 (1:1000; Cell Signaling Technology), anti-TBLR1 (1:1000; Abcam, Cambridge, United Kingdom), anti-HA (1:2000; Proteintech, Wuhan, China), anti-β-actin (1:5000; Cell Signaling Technology). Secondary antibodies included donkey anti-rabbit (1:2500; Cell Signaling Technology), rabbit anti-mouse (1:2500; Cell Signaling Technology), and anti-light chain (1:3000; Abcam).

### Ubiquitination and CHX-Protein Stability Assays

For the ubiquitination assay, HA-ubiquitin plasmids were transfected into USP1-NC and USP1-KO cells. Following treated with the proteasome inhibitor MG-132 (5 μM) for 6 h, cells were harvested and lysed for immunoprecipitation of TBLR1 and immunoblotting of HA. Western blot was performed as described above. For the CHX-protein stability assay, in order to inhibit protein synthesis, cells in each group were treated with CHX (100 μg/mL) for 0, 3, 6, and 9 h. MG-132 (5 μM) was added along with CHX (16). Cell lysates were collected and western blot was performed as described above.

### Establishment of Mouse Tumor Xenograft Model

Twenty male BALB/c nude mice were divided into four groups randomly (*n* = 5/group). For the subcutaneous assay, 1 × 10^6^ tumor cells were injected subcutaneously into each mouse. For the liver xenograft assay, 1 × 10^6^ tumor cells were transplanted into the hepatic lobes of mice. All animal experiments were approved by the Research Ethics Committee of the Zhongshan Hospital (B2017-159R) and mice were sacrificed at 5 weeks post-injection.

### Statistical Analysis

All experimental results were obtained from assays performed in triplicate and are shown as the mean ± standard deviation. Relationships between *USP1* expression and clinicopathological factors were analyzed using the Pearson χ^2^ test. Differences between treated and control groups were determined using the Student’s *t*-test and one-way analysis of variance. *P* < 0.05 was considered a statistically significant result.

## Results

### USP1 Is Upregulated in HCC and Correlated With Metastasis

**FIGURE 1:**
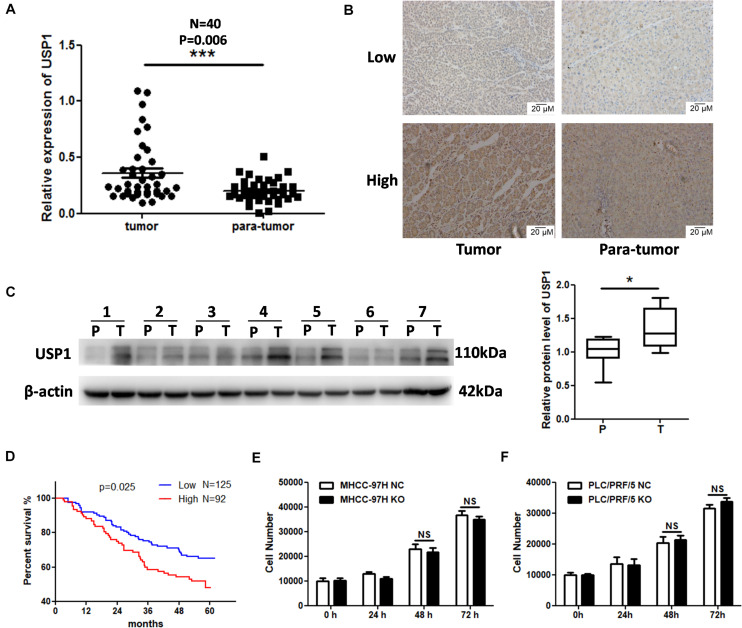
Elevated expression of *USP1* in HCC. **(A)** USP1 mRNA levels in 40-paired specimens of HCC tissues and matched para-tumor liver tissues. **(B)** IHC staining of USP1 in 56 pairs of HCC and matching para-tumor tissues. Representative images are shown. **(C)** USP1 levels in seven paired samples. T, HCC tissues; P, para-tumor tissues. β-actin was used as a control. **(D)** Kaplan–Meier analysis with log-rank testing of survival was performed in 217 patients with HCC exhibiting different *USP1* expression levels. Error bars represent the standard deviation. *P = 0.025. **(E,F)** Cell numbers were count by cell counting instrument after 0, 24, 48, and 96 h of seeding.

Ubiquitin-specific protease 1 is ubiquitously expressed in human tissues. We first assessed *USP1* mRNA levels in tumor and para-tumor tissues, which revealed higher *USP1* expression in tumor tissues than in para-tumor tissues (Figure 1A). IHC and western blot analysis also confirmed higher levels of USP1 in HCC samples relative to those in adjacent non-tumor tissues (Figures 1B,C). Next, a tissue microarray with 217 samples of HCC tissues was IHC stained to test the correlations between USP1 levels and overall survival (OS) of patients. According to the staining intensity, we observed elevated levels of USP1 (92/217) in patients with a short OS and low levels (125/217) in patients with a long OS (*P* = 0.0248) (Figure 1D). We then evaluated the relationship between the USP1 levels and the clinical characteristics of patients with HCC (Supplementary Table S1). Interestingly, the only clinical characteristics positively correlated with USP1 levels were serum α-fetoprotein level (*P* = 0.013) and tumor number (*P* = 0.028). USP1 level was not correlated with tumor size (*P* = 0.696). These results indicates that the short OS of HCC patients with high levels of USP1 is mainly caused by metastasis rather than proliferation.

We knocked down the expression of USP1 in MHCC-97H cells (Supplementary Figure S1A) and, as expected, found no significant difference in proliferation between USP1-knockdown cells and control cells (Supplementary Figure S1B). Further, we created USP1 knockout (USP1-KO) cells in MHCC-97H and PLC/PRF/5 cell lines by sgRNA-Cas9 system (Supplementary Figure S1C). The data showed USP1 level did not correlate with proliferation (Figures 1E,F). These results indicated that USP1 is involved in metastasis and may be the reason for poor patient prognoses. We found no significant difference in migration or invasion between USP1-knockdown cells and control cells (Supplementary Figures S1D,E). Thus, we hypothesized that USP1 contributed to cancer metastasis mainly by promoting cancer cell survival in the blood rather than promoting cancer cell invasion.

### USP1 Maintains CTC Survival in Blood-Borne Metastasis

**FIGURE 2:**
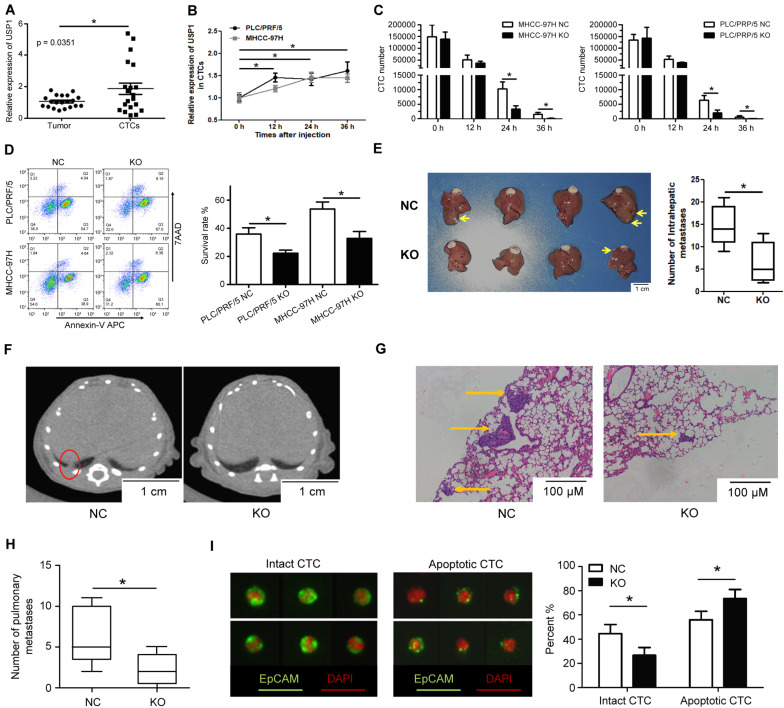
USP1 promotes circulating tumor cell survival in blood. **(A)** *USP1* mRNA expression level in primary tumors and CTCs. **(B)** *USP1* expression at 0, 12, 24, and 36 h after tail injection. **(C)** After 0, 12, 24, and 36 h of tail injection, GFP-labeled cells were sorted and counted by FACS. **(D)** 24 h after injection, GFP-labeled cells were sorted and labeled with Annexin V-APC and 7AAD. **(E)** Intrahepatic metastatic tumors in hepatic lobes 5 weeks after liver xenografting. **(F,G)** Lung metastasis detected by computed tomography and IHC. The arrows were used to show lung metastasis. **(H)** Bar graph of pulmonary metastases tumor numbers. **(I)** CTCs detected in blood and labeled by EpCAM and pan-cytokeratin (green) and DAPI (red). *P < 0.05.

To identify the role of USP1 in cancer cell survival in the blood, we obtained single CTCs from peripheral vein blood and extracted RNA using a single-cell-to-CT quantitative reverse transcription-PCR (qRT-PCR) kit. We observed that *USP1* expression in CTCs was higher than in primary tumor cells (Figure 2A). Additionally, we injected PLC/PRF/5-GFP or MHCC-97H-GFP cells into the peripheral tail vein of nude mice and sorted GFP-positive cells by flow cytometry after 0, 12, 24, and 36 h, with results showing that *USP1* expression increased over time (Figure 2B). These results indicate USP1 is involved in the survival of CTCs.

To test the above hypothesis, cancer cells (1 × 10^6^) were injected into mice via the peripheral tail vein, which simulated CTCs in the blood. FACS cell counting results showed that the number of CTCs in the *USP1*-KO group was less than in the control group (Figure 2C). Apoptotic assays showed that *USP1*-KO cells have a lower survival rate compared with control cells at 24 h post-injection (Figure 2D). Similar results were observed in USP1-knockdown cells and control cells (Supplementary Figure S2A). Individually *USP1* knockout did not affect cell apoptosis in cultured medium (Supplementary Figure S2B). These results indicated that USP1 depletion attenuated the survival ability of CTCs.

A Xenograft-formation assay was performed to determine the effects of USP1 *in vivo*. After 5 weeks of liver xenografting, the *USP1*-KO group showed a lower tumor number than the *USP1*-NC group (*P* < 0.05) (Figure 2E). Furthermore, we observed pulmonary tumor formation in the control group but not in the *USP1*-KO group according to computed tomography (Figure 2F) and hematoxylin-eosin staining (Figures 2G,H). Additionally, apoptotic cell usually exhibit the pattern with cell shrinkage or cell membrane rupture which can be detected by cell surface marker (17). We enriched the CTCs in mouse blood, labeled the CTCs with fluorescent EpCAM (green), detected CTCs with their apoptotic pattern^16^ by microscope (Figure 2I, left) and found that knockout of USP1 increased apoptotic-like CTC patterns compared with the control group (USP1-NC 55% VS USP1-KO 73%) (Figure 2I, right). These observations demonstrate that USP1 modulates HCC CTC blood-borne metastasis *in vivo*. Moreover, the cell apoptotic rate (Figure 2D) and metastases number (Figure 2H) confirmed that USP1 assists CTC survival in the bloodstream.

### USP1 KO Impairs Wnt Targets in HCC

**FIGURE 3:**
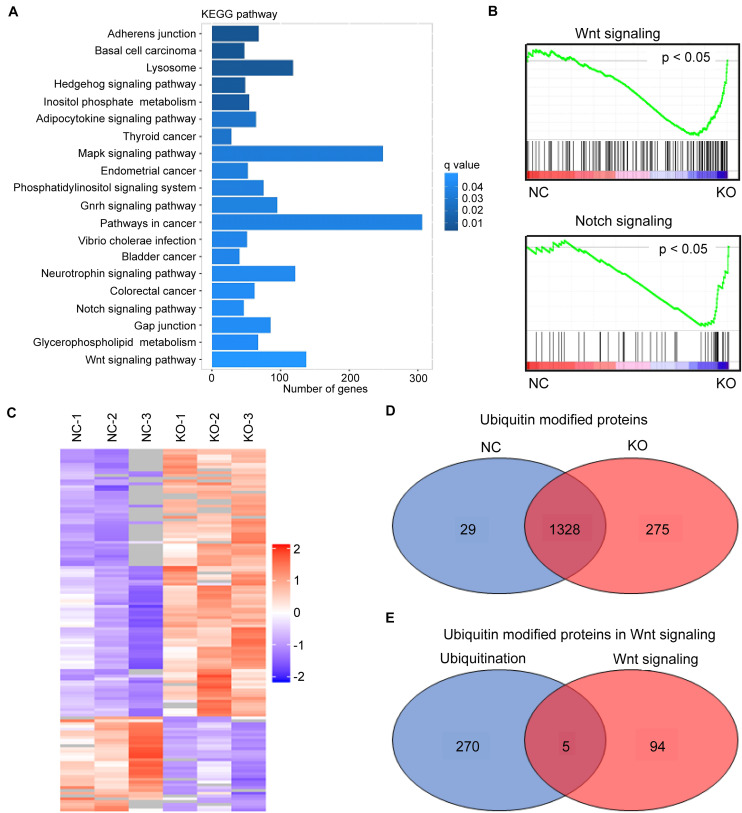
*USP1* KO impairs Wnt targets. **(A)** KEGG pathway enrichment of differentially expressed genes (DEGs) in *USP1*-KO cells versus control cells (in MHCC-97H cell line). **(B)** GSEA enrichment plots of Wnt signaling and Notch signaling. **(C)** Identification of USP1-deubiquitinating targets using a combination of ubiquitin-chain-specific IP and label-free LC-MS/MS analysis. **(D)** Venn diagram showing the number of ubiquitin-modified proteins in the two groups. **(E)** Venn diagram showing five overlapping proteins between the *USP1*-KO group and Wnt signaling.

High-throughput sequencing (The Beijing Genomics Institute, Beijing, China) was used to identify USP1-regulated pathways in HCC and determine how USP1 affects cancer cell survival and metastasis. As expected, compared with those in the *USP1*-KO group, the Kyoto Encyclopedia of Genes and Genomes (KEGG) analysis showed the enrichment of Wnt, Notch, and Hedgehog pathways, which play essential roles in cancer stem cell regulation (18) and may support the survival of CTCs (Figure 3A). In gene set enrichment analysis, Wnt signaling and Notch signaling showed significant associations with USP1 knockout (Figure 3B).

The inhibition of most Wnt targets by *USP1* KO was confirmed by real-time PCR (Supplementary Figure S3). These results suggest USP1 as an essential factor involved in Wnt-signaling. We performed a label-free ubiquitin quantitative assay using MHCC-97H cells to determine the USP1 target(s) in the Wnt signaling pathway. Ubiquitin is the substrate of deubiquitinases; therefore, immunoprecipitation (IP) with an antibody against ubiquitin chains can enrich ubiquitin-modified proteins. Moreover, subsequent high-throughput liquid chromatography-tandem mass spectrometry (LC-MS/MS) analysis (Shanghai Applied Protein Technology Company, Shanghai, China) and quantitative proteomics analysis were performed to investigate how USP1 influences the ubiquitination levels of the target peptide-binding region in HCC cell lines (Figure 3C). Cells were divided into two groups: the MHCC-97H-*USP1*-NC cell line with endogenous USP1-deubiquitinating activity and MHCC-97H-*USP1-*KO cell line with no USP1-deubiquitinating activity. Among the peptide-matched proteins, 29 proteins were identified as part of the ubiquitin interactome only in MHCC-97H-*USP1*-NC cells (Figure 3D) and not in USP1-KO cells, whereas 275 highly ubiquitin modified proteins were specifically detected in *USP1*-KO cells, indicating that USP1 can modulate the cell state by deubiquitinating these proteins. KEGG analysis showed that among these 275 proteins, five participate in Wnt signaling, including TBLR1, Ras-related C3 botulinum toxin substrate 1, SMAD4, BMP4 and protein phosphatase 2B regulatory subunit 1 (Figure 3E).

### USP1 Maintains the Survival of CTCs by Deubiquitinating and Stabilizing TBLR1

**FIGURE 4:**
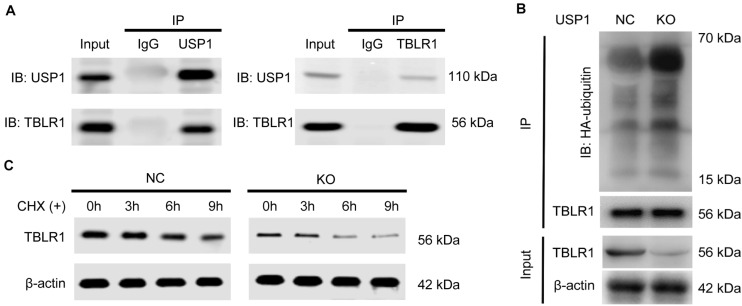
USP1 interacts, deubiquitinates, and stabilizes TBLR1. **(A)** Co-IP assays of USP1 and TBLR1 in MHCC-97H cells. **(B)** Impact of USP1 on TBLR1 ubiquitination *in vivo*. Immunoblot using an HA-tag to detect poly-ubiquitination of TBLR1. **(C)** USP1 enhances TBLR1 stability; cells were treated with CHX (100 μg/mL) and collected at 0, 3, 6, and 9 h. TBLR1 levels were analyzed by western blotting.

We then investigated the biochemical interaction between USP1 and these five proteins. We observed that USP1 interacted with TBLR1 in a Co-IP assay (Figure 4A). Reciprocal Co-IP assays using tag antibodies revealed similar results, showing that USP1 can co-interact with TBLR1 (Supplementary Figure S4A). In contrast, the other four proteins did not show a co-interaction with USP1 (Supplementary Figure S4B). *USP1* KO decreased TBLR1 protein levels in PLC/PRF/5 and MHCC-97H cells (Supplementary Figure S4C); however, *TBLR1* mRNA levels were unaffected by *USP1* KO (Supplementary Figure S4D). Additionally, in patient samples, we found that USP1 was positively correlated with the TBLR1 level at the protein but not the mRNA level (Supplementary Figure S4E). Moreover, we observed that PLC/PRF/5 and MHCC-97H cells degraded TBLR1 in a proteasome-dependent manner, as TBLR1 accumulated after treatment with the proteasome inhibitor MG-132 (Supplementary Figure S4F). These findings demonstrate that USP1 regulates TBLR1 at the protein level. We performed an *in vitro* ubiquitination assay to determine whether USP1 stabilizes TBLR1 in a deubiquitination-dependent manner. Using an anti-TBLR1 antibody for co-IP, we showed that in the absence of USP1, the HA-ubiquitin ligation level was enhanced (Figure 4B). Furthermore, we conducted a cycloheximide (CHX) chase assay to investigate the effects of USP1 on TBLR1 stability. After 0, 3, 6, and 9 h of CHX treatment, TBLR1 levels were quantified by western blot analysis (Figure 4C). As expected, TBLR1 degradation occurred faster in *USP1-*KO cells than in control cells. These results demonstrated that USP1 deubiquitinates and stabilizes TBLR1.

**FIGURE 5:**
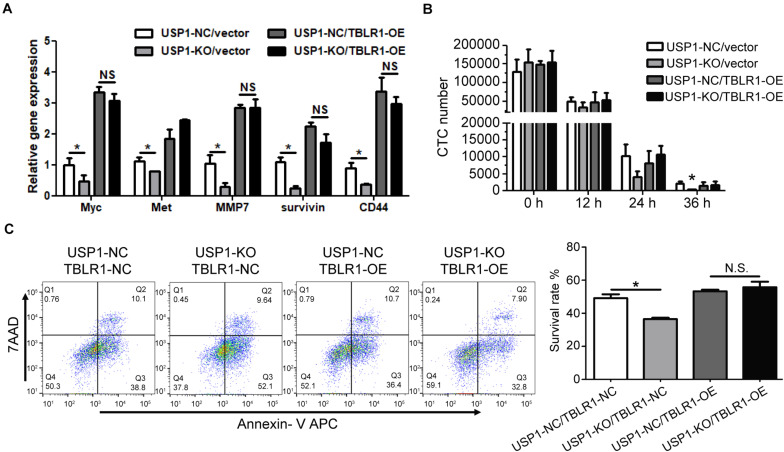
Overexpressing TBLR1 rescues the survival of CTC by USP1 depletion. **(A)** TBLR1 overexpression rescued the expression of Wnt targets caused by USP1-knockout. **(B,C)** Constructed from the MHCC-97H cell line, USP1-NC-GFP-vector cells, USP1-KO-GFP-vector cells, USP1-NC-GFP-TBLR1-OE cells, and USP1-KO-GFP-TBLR1-OE cells were injected into the peripheral tail vein. After 24 h, the CTC number **(B)** and cell-survival rate **(C)** was detected by FACS. The CTC number **(B)** and cell-survival rate **(C)** of USP1-NC-GFP-vector cells, USP1-KO-GFP-vector cells, USP1-NC-GFP-TBLR1-OE cells, and USP1-KO-GFP-TBLR1-OE cells after 24 h of tail injection. *P < 0.05.

**FIGURE 6:**
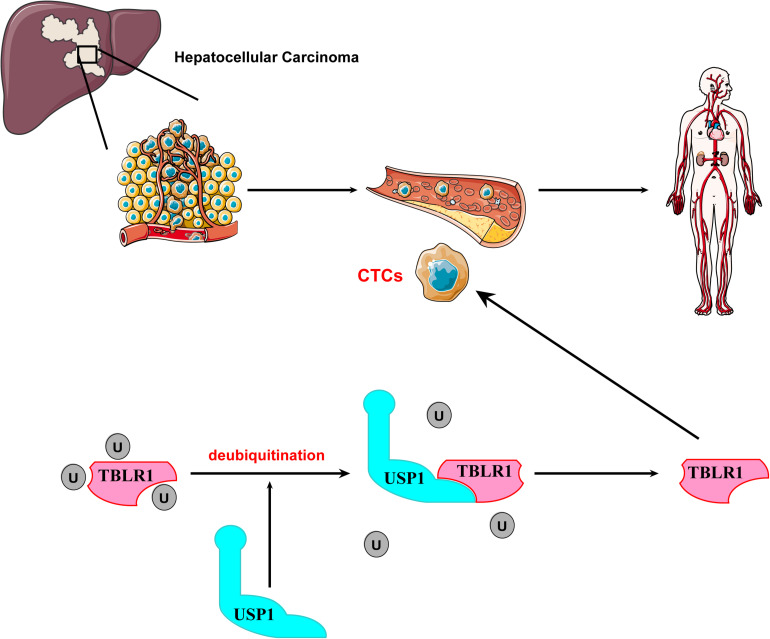
The mechanism of USP1 maintaining the survival of CTCs by stabilizing TBLR1.

The depletion of TBL1X-TBLR1 significantly inhibited the expression of Wnt target genes (19, 20). To show that USP1 regulates Wnt signaling by deubiquitinating TBLR1 in HCC, we overexpressed *TBLR1* (TBLR1-OE). Compared with control cells, TBLR1-OE cells had increased levels of c-Myc, Met, MMP7, and CD44 (Figure 5A), and overexpression rescued the repression effect of USP1 depletion. After overexpressing TBLR1, USP1-NC-TBLR1-OE cells, and USP1-NC-TBLR1-OE cells showed the similar HA-ubiquitin ligation level, which means overexpressing TBLR1 rescued the ubiquitination effect of USP1 depletion (Supplementary Figure S5). Besides, overexpressing *TBLR1*, *USP1*-KO and control cells showed a similar cell count number (Figure 5B) and survival rate (Figure 5C) at 24 h after injection via the tail vein. These results demonstrate that USP1 maintains the survival of CTCs by stabilizing TBLR1 (Figure 6).

## Discussion

Recent studies of CTCs have mostly focused on the relationship between CTC counts and clinical patterns (21); however, studies of the mechanisms underlying CTC survival are limited. We investigated the CTC survival mechanism by gene profiling, proteomics analysis, and analyzing changes in signaling pathways. The results suggested that USP1 promotes CTC survival, which may lead to metastasis and recurrence. We also prepared *USP1*-overexpressing PLC/PRF/5 cells and MHCC-97H cells; however, we found no significant changes in phenotypes, such as CTC apoptosis, proliferation, migration, colony formation, and spheroid formation, between *USP1*-NC and *USP1*-overexpressing cells. We hypothesized that USP1 is regulated by signal molecules when deubiquitination is required rather than diffused in the cytoplasm for random substrate deubiquitination.

Cancer metastasis is an inefficient process, with only a small proportion of tumor cells successfully surviving hematogenous spreading (22). CTCs should endure various forms of stress such as anoikis, reactive oxygen species, chemotherapy drugs, fluid shear stress, the immune system, and senescence during blood-borne metastasis. Adjusting to the specialized microenvironment, adult stem cells may regulate their state such as proliferation, quiescence, self-renewal, or differentiation (23, 24). Cancer stem cells arise from mutant stem cells, which may benefit from the transformation of suitable cell state to fit different microenvironments (24, 25). It has been reported that CTCs with stem cell characteristics are at higher risk for tumor recurrence and metastasis (26). In this study, RNA-seq results showed the enrichment of 3 stem cell-related signaling in the top 20 pathways, including Wnt signaling, Notch signaling, and Hedgehog signaling, indicating a critical role for USP1 in cancer cell stemness. As Notch signaling plays an essential role in cancer stem cells, we detected downregulation of *Notch1* and *Notch2* in *USP1*-KO cells by qRT-PCR; however, we did not detect any difference in ubiquitination of proteins involved in Notch signaling between *USP1-*KO and wild-type cells using label-free quantification LC-MS/MS. Therefore, the mechanism of action of USP1 involving Notch signaling requires further investigation. As an aspect of cancer stem cell and metastasis, a previous study, which reported that USP1 preserves osteosarcoma stem cells by deubiquitinating ID proteins (12) also observed a relationship between USP1 and circulating tumor stem cells. Thus, whether USP1 maintains the survival of CTCs by preserving CTCs in a stem cell-like state should be further investigated.

Ubiquitin-specific protease 1 could not directly affect the migration or invasion of cancer cells (Supplementary Figures S1D,E), whereas adherent junction pathways can still be enriched in RNA-seq. Moreover, we often observe evidence of anchorage dependence in our recent CTC research (15). We consider that an anchorage-dependent microenvironment plays a crucial role in CTC survival; however, the mechanism is unclear. Platelets are known to affect the CTC microenvironment (27). Platelets may be recruited and surrounded by CTCs to shield and provide an anchoring base for the CTCs, avoid anoikis, avoid damage caused by fluid shear stress, and protect CTCs from immune cell attack (28, 29). Drugs that can neutralize the microenvironment of CTCs in the blood may be useful for attenuating metastases. We found that USP1-specific inhibitors, such as SJB3, could affect the survival of CTCs. Our future studies will focus on the mechanism of USP1 inhibitors in the blood-borne survival of CTCs.

Attack by the immune system is a major factor limiting the survival of CTCs. Some CTCs may enter a dormant state to evade immune surveillance, whereas others may upregulate their “do not eat me” signals to enable them to escape from the immune system (23, 30, 31). We co-cultured USP1-KO cells or USP1-NC cells with immune cells (CD8+ T cells or natural killer cells); however, our evidence is insufficient to demonstrate a relationship between USP1 and immune escape.

We also established a CTC blood-borne mouse model to investigate the relationship between visible CTC survival CTC genotype states; however, because of the limitations associated with CTC acquisition and culture, we were unable to investigate real-time changes in patient CTCs, which will prevent the development of personalized therapy. CTC survival is the primary cause of metastasis, suggesting that inhibition of USP1, a potential therapeutic target, can effectively induce the apoptosis of CTCs in the blood and reduce metastasis. Additionally, numerous chemicals and target-directed drugs are being used to induce apoptosis in cancer cells. Evaluation of the CTC survival rate and genotype after treatment with anticancer reagents may be a practical approach for determining disease prognosis, which may also accelerate the development of novel therapeutics.

## Data Availability Statement

The raw data supporting the conclusions of this article will be made available by the authors, without undue reservation.

## Ethics Statement

The human and animal study was approved by the Research Ethics Committee of the Zhongshan Hospital (B2017-159R). The patients/participants provided their written informed consent to participate in this study. The animal study was approved by the Research Ethics Committee of the Zhongshan Hospital (B2017-159R).

## Author Contributions

YL, YX, CG, and XY participated in the conception and design of the study. YL, CG, YS, KZ, PW, WG, and JF performed the experiments. YL, YX, CG, JC, CY, JF, and XY interpreted the data produced and edited the drafts of the manuscript. All authors contributed to the article and approved the submitted version.

## Conflict of Interest

The authors declare that the research was conducted in the absence of any commercial or financial relationships that could be construed as a potential conflict of interest.

## Supplementary Material

FIGURE S1 Migration and invasion ability of USP1 knockdown cells. **(A)** USP1 knockdown cells were constructed in the MHCC-97H cell line. The *USP1* expression level was measured by qPCR. **(B)** 1 × 10^4^ cells were seed in a 96 well plate. Cell numbers were count using a cell counting instrument after 0, 24, 48, and 96 h of seeding. **(C)** The *USP1* knockout efficiency in PLC/PRF/5 and MHCC-97H cell lines. **(D,E)** Wound healing assay and Transwell assay using *USP1*-NC cells and *USP1*-KO cells in the MHCC-97H cell line.

FIGURE S2 USP1 deficiency inhibits CTC survive and tumor growth. **(A)** USP1-NC-GFP and USP1-KD-GFP cells were injected into the peripheral tail vein. After 24 h, GFP-cells were sorted by FACS and labeled by Annexin V-APC and 7AAD. **(B)** Apoptosis rate of USP1 knockout cells and control cells.

FIGURE S3 mRNA levels of Wnt targets in NC cell lines and *USP1*-KO cell lines.

FIGURE S4 USP1 interacts with TBLR1 and correlates with TBLR1 protein level. **(A)** Co-IP assays of USP1-HA and TBLR1-Flag in 293T cells. **(B)** Co-IP assays of USP1-HA and RAC1, SMAD4, BMP4 or PPP2R1 in 293T cells. **(C)** TBLR1 expression in USP1-NC and USP1-KO cell lines. **(D)** TBLR1 mRNA level in USP1-NC and USP1-KO cell lines. **(E)** Correlation of USP1 and TBLR1 in patient samples. **(F)** TBLR1 is degraded in a proteasome-dependent manner and inhibited by MG-132.

FIGURE S5 Ub assay in overexpression system by using USP1, TBLR1, and Ub plasmid.

TABLE S1 Expression of USP1 and correlation with clinical characteristics of HCC patients (*n* = 217).

## References

[1] Siegel RL Miller KD Jemal A. Cancer statistics, 2020. *CA Cancer J Clin.* (2020) 70:7–30. 10.3322/caac.21590 31912902

[2] Cha C Fong Y Jarnagin WR Blumgart LH DeMatteo RP. Predictors and patterns of recurrence after resection of hepatocellular carcinoma. *J Am College Surg.* (2003) 197:753–8. 10.1016/j.jamcollsurg.2003.07.003 14585409

[3] Forner A Reig M Bruix J. Hepatocellular carcinoma. *Lancet.* (2018) 391:1301–14. 10.1016/S0140-6736(18)30010-2 29307467

[4] Chaffer CL Weinberg RA. A perspective on cancer cell metastasis. *Science.* (2011) 331:1559–64. 10.1126/science.1203543 21436443

[5] Williams SC. Circulating tumor cells. *Proc Natl Acad Sci USA.* (2013) 110:4861. 10.1073/pnas.1304186110 23533270 PMC3612640

[6] Lianidou ES Strati A Markou A. Circulating tumor cells as promising novel biomarkers in solid cancers. *Crit Rev Clin Lab Sci.* (2014) 51:160–71. 10.3109/10408363.2014.896316 24641350

[7] Vucic D Dixit VM Wertz IE. Ubiquitylation in apoptosis: a post-translational modification at the edge of life and death. *Nat Rev Mol Cell Biol.* (2011) 12:439–52. 10.1038/nrm3143 21697901

[8] Nakayama KI Nakayama K. Ubiquitin ligases: cell-cycle control and cancer. *Nat Rev Cancer.* (2006) 6:369–81. 10.1038/nrc1881 16633365

[9] Geng F Wenzel S Tansey WP. Ubiquitin and proteasomes in transcription. *Annu Rev Biochem.* (2012) 81:177–201. 10.1146/annurev-biochem-052110-120012 22404630 PMC3637986

[10] Schaefer A Nethe M Hordijk PL. Ubiquitin links to cytoskeletal dynamics, cell adhesion and migration. *Biochem J.* (2012) 442:13–25. 10.1042/BJ20111815 22280013

[11] Ulrich HD Walden H. Ubiquitin signalling in DNA replication and repair. *Nat Rev Mol Cell Biol.* (2010) 11:479–89. 10.1038/nrm2921 20551964

[12] Williams SA Maecker HL French DM Liu J Gregg A Silverstein LB USP1 deubiquitinates ID proteins to preserve a mesenchymal stem cell program in osteosarcoma. *Cell.* (2011) 146:918–30. 10.1016/j.cell.2011.07.040 21925315

[13] Jung JK Jang SW Kim JM. A novel role for the deubiquitinase USP1 in the control of centrosome duplication. *Cell Cycle.* (2016) 15:584–92. 10.1080/15384101.2016.1138185 26822809 PMC5056607

[14] Ran FA Hsu PD Wright J Agarwala V Scott DA Zhang F. Genome engineering using the CRISPR-Cas9 system. *Nat Protoc.* (2013) 8:2281–308. 10.1038/nprot.2013.143 24157548 PMC3969860

[15] Sun YF Guo W Xu Y Shi YH Gong ZJ Ji Y Circulating tumor cells from different vascular sites exhibit spatial heterogeneity in epithelial and mesenchymal composition and distinct clinical significance in hepatocellular carcinoma. *Clin Cancer Res.* (2018) 24:547–59. 10.1158/1078-0432.CCR-17-1063 29070526

[16] Nguyen TTT Park EM Lim YS Hwang SB. Nonstructural protein 5A impairs DNA damage repair: implications for hepatitis C virus-mediated hepatocarcinogenesis. *J Virol.* (2018) 92:e00178-18. 10.1128/JVI.00178-18 29563287 PMC5952158

[17] Larson CJ Moreno JG Pienta KJ Gross S Repollet M O’Hara SM Apoptosis of circulating tumor cells in prostate cancer patients. *Cytometry A.* (2004) 62:46–53. 10.1002/cyto.a.20073 15472900

[18] Wang R Sun Q Wang P Liu M Xiong S Luo J Notch and Wnt/beta-catenin signaling pathway play important roles in activating liver cancer stem cells. *Oncotarget.* (2016) 7:5754–68. 10.18632/oncotarget.6805 26735577 PMC4868719

[19] Perissi V Scafoglio C Zhang J Ohgi KA Rose DW Glass CK TBL1 and TBLR1 phosphorylation on regulated gene promoters overcomes dual CtBP and NCoR/SMRT transcriptional repression checkpoints. *Mol Cell.* (2008) 29:755–66. 10.1016/j.molcel.2008.01.020 18374649 PMC2364611

[20] Hoberg JE Yeung F Mayo MW. SMRT derepression by the IkappaB kinase alpha: a prerequisite to NF-kappaB transcription and survival. *Mol Cell.* (2004) 16:245–55. 10.1016/j.molcel.2004.10.010 15494311

[21] Ruscetti M Quach B Dadashian EL Mulholland DJ Wu H. Tracking and functional characterization of epithelial-mesenchymal transition and mesenchymal tumor cells during prostate cancer metastasis. *Cancer Res.* (2015) 75:2749–59. 10.1158/0008-5472.CAN-14-3476 25948589 PMC4490048

[22] Wong CW Lee A Shientag L Yu J Dong Y Kao G Apoptosis: an early event in metastatic inefficiency. *Cancer Res.* (2001) 61:333–8. 11196183

[23] Massague J Obenauf AC. Metastatic colonization by circulating tumour cells. *Nature.* (2016) 529:298–306. 10.1038/nature17038 26791720 PMC5029466

[24] Oskarsson T Batlle E Massague J. Metastatic stem cells: sources, niches, and vital pathways. *Cell Stem Cell.* (2014) 14:306–21. 10.1016/j.stem.2014.02.002 24607405 PMC3998185

[25] Driessens G Beck B Caauwe A Simons BD Blanpain C. Defining the mode of tumour growth by clonal analysis. *Nature.* (2012) 488:527–30. 10.1038/nature11344 22854777 PMC5553110

[26] Yamashita T Wang XW. Cancer stem cells in the development of liver cancer. *J Clin Invest.* (2013) 123:1911–8. 10.1172/JCI66024 23635789 PMC3635728

[27] Mammadova-Bach E Mangin P Lanza F Gachet C. Platelets in cancer. From basic research to therapeutic implications. *Hamostaseologie.* (2015) 35:325–36. 10.5482/hamo-14-11-0065 26289826

[28] Palumbo JS Talmage KE Massari JV La Jeunesse CM Flick MJ Kombrinck KW Platelets and fibrin(ogen) increase metastatic potential by impeding natural killer cell-mediated elimination of tumor cells. *Blood.* (2005) 105:178–85. 10.1182/blood-2004-06-2272 15367435

[29] Labelle M Hynes RO. The initial hours of metastasis: the importance of cooperative host-tumor cell interactions during hematogenous dissemination. *Cancer Discov.* (2012) 2:1091–9. 10.1158/2159-8290.CD-12-0329 23166151 PMC3540992

[30] Lian S Xie R Ye Y Lu Y Cheng Y Xie X Dual blockage of both PD-L1 and CD47 enhances immunotherapy against circulating tumor cells. *Sci Rep.* (2019) 9:4532. 10.1038/s41598-019-40241-1 30872703 PMC6418176

[31] Steinert G Scholch S Niemietz T Iwata N Garcia SA Behrens B Immune escape and survival mechanisms in circulating tumor cells of colorectal cancer. *Cancer Res.* (2014) 74:1694–704. 10.1158/0008-5472.CAN-13-1885 24599131

