# Supplementary figures and images for: USP1 Maintains the Survival of Liver Circulating Tumor Cells by Deubiquitinating and Stabilizing TBLR1
Source: Front Oncol. 2020 Sep 25;10:554809. doi: 10.3389/fonc.2020.554809 (PMC7545832; doi:10.3389/fonc.2020.554809)

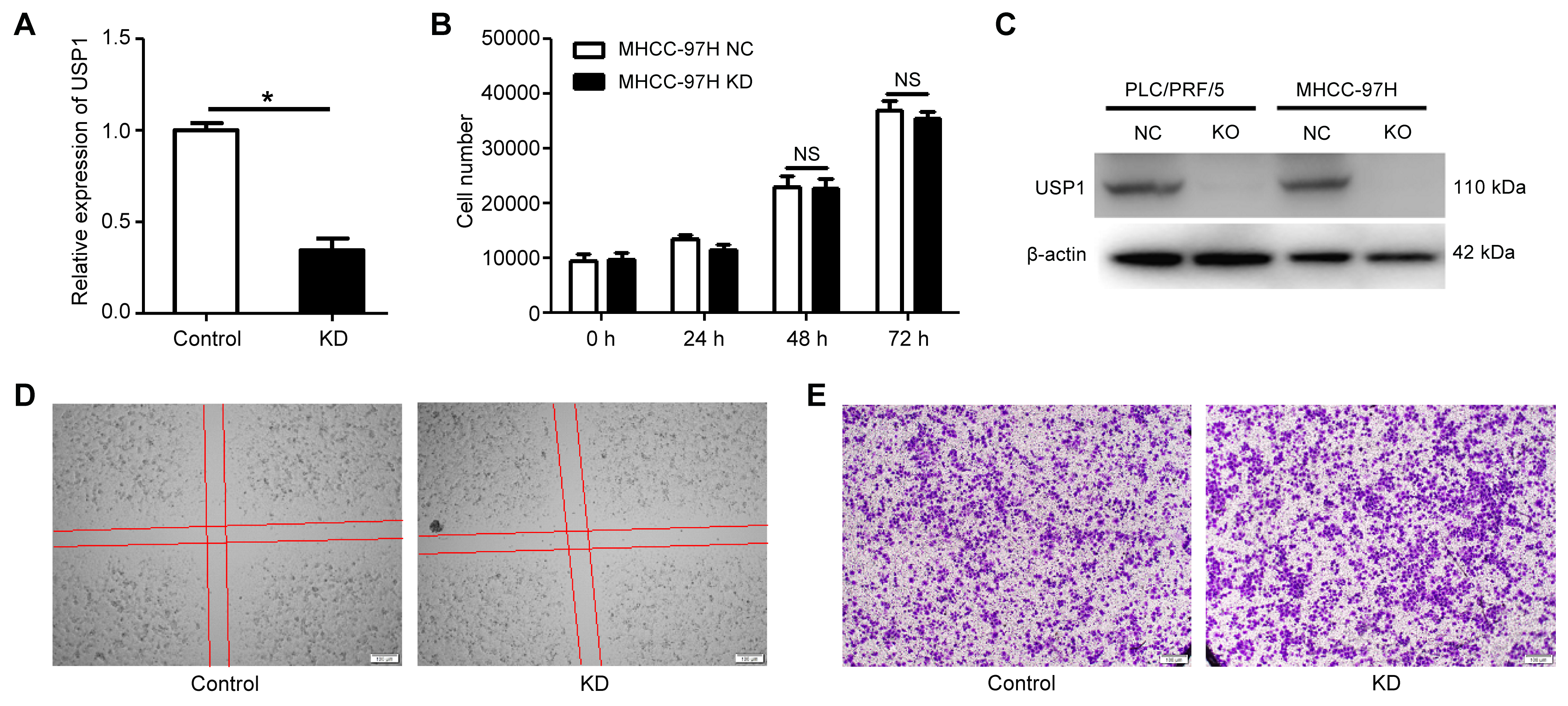

Supplement: FIGURE S1 — Migration and invasion ability of USP1 knockdown cells. (A) USP1 knockdown cells were constructed in the MHCC-97H cell line. The USP1 expression level was measured by qPCR. (B) 1 × 10^4^ cells were seed in a 96 well plate. Cell numbers were count using a cell counting instrument after 0, 24, 48, and 96 h of seeding. (C) The USP1 knockout efficiency in PLC/PRF/5 and MHCC-97H cell lines. (D,E) Wound healing assay and Transwell assay using USP1-NC cells and USP1-KO cells in the MHCC-97H cell line. [file Image_1.TIF]

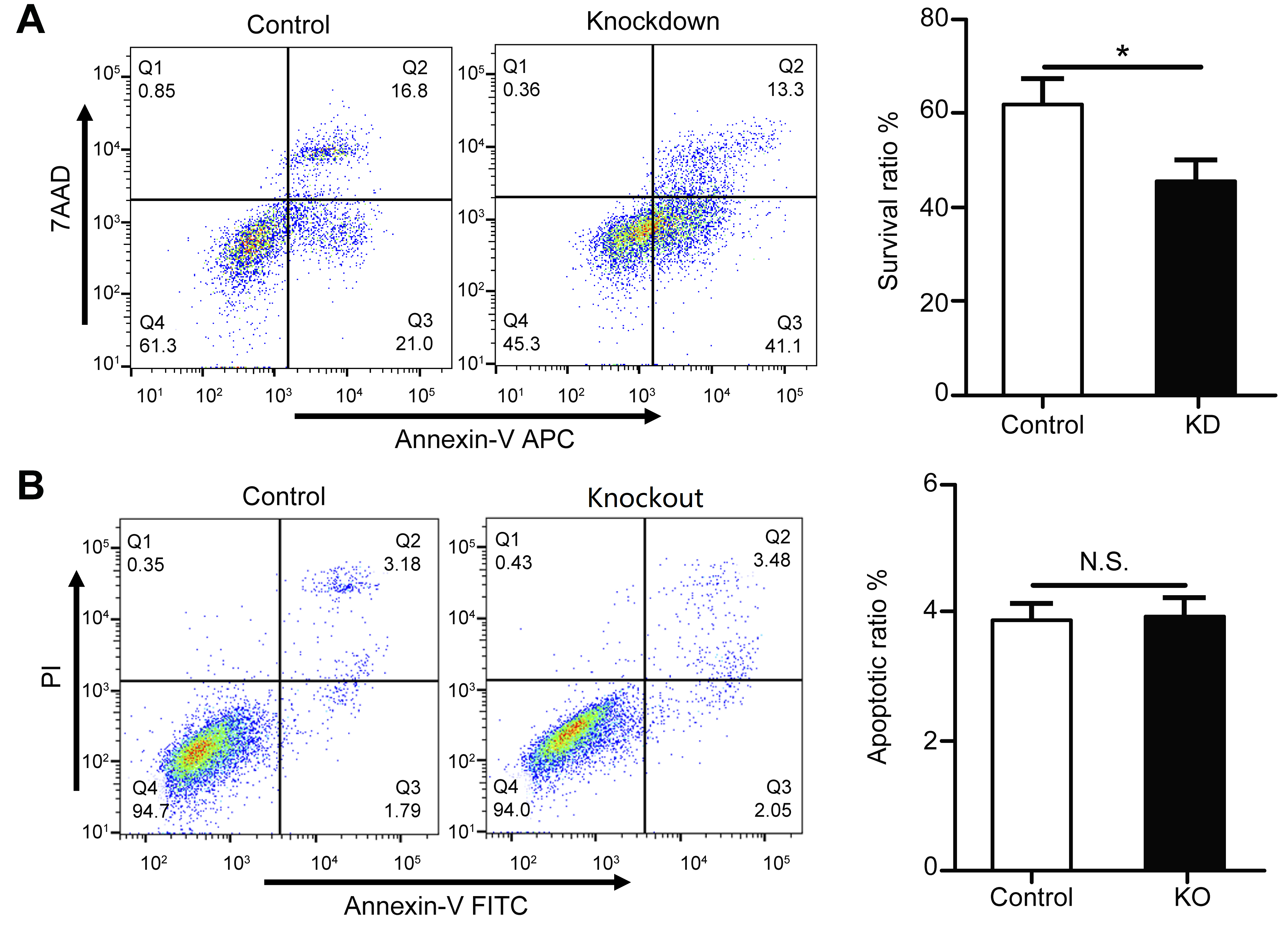

Supplement: FIGURE S2 — USP1 deficiency inhibits CTC survive and tumor growth. (A) USP1-NC-GFP and USP1-KD-GFP cells were injected into the peripheral tail vein. After 24 h, GFP-cells were sorted by FACS and labeled by Annexin V-APC and 7AAD. (B) Apoptosis rate of USP1 knockout cells and control cells. [file Image_2.TIF]

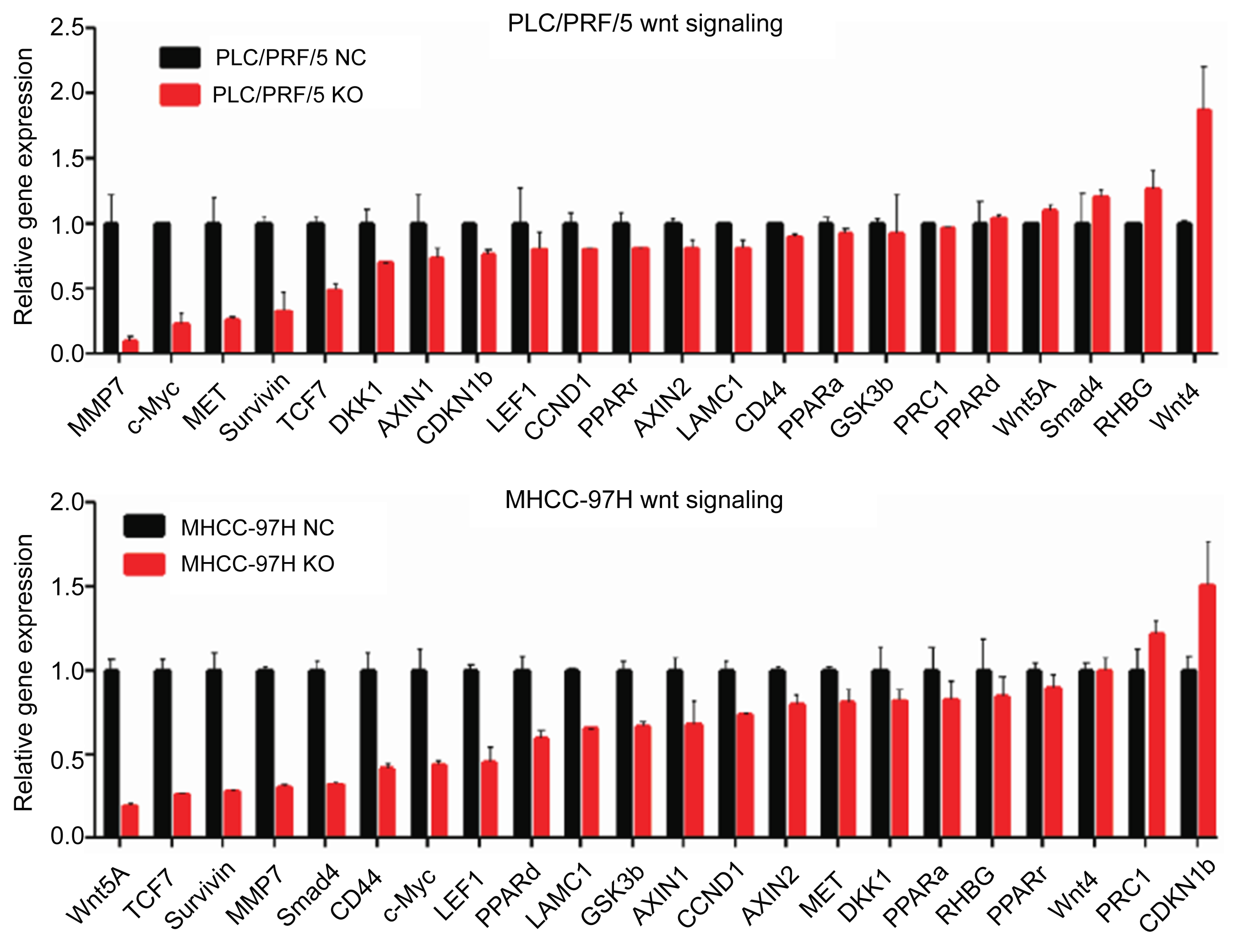

Supplement: FIGURE S3 — mRNA levels of Wnt targets in NC cell lines and USP1-KO cell lines. [file Image_3.TIF]

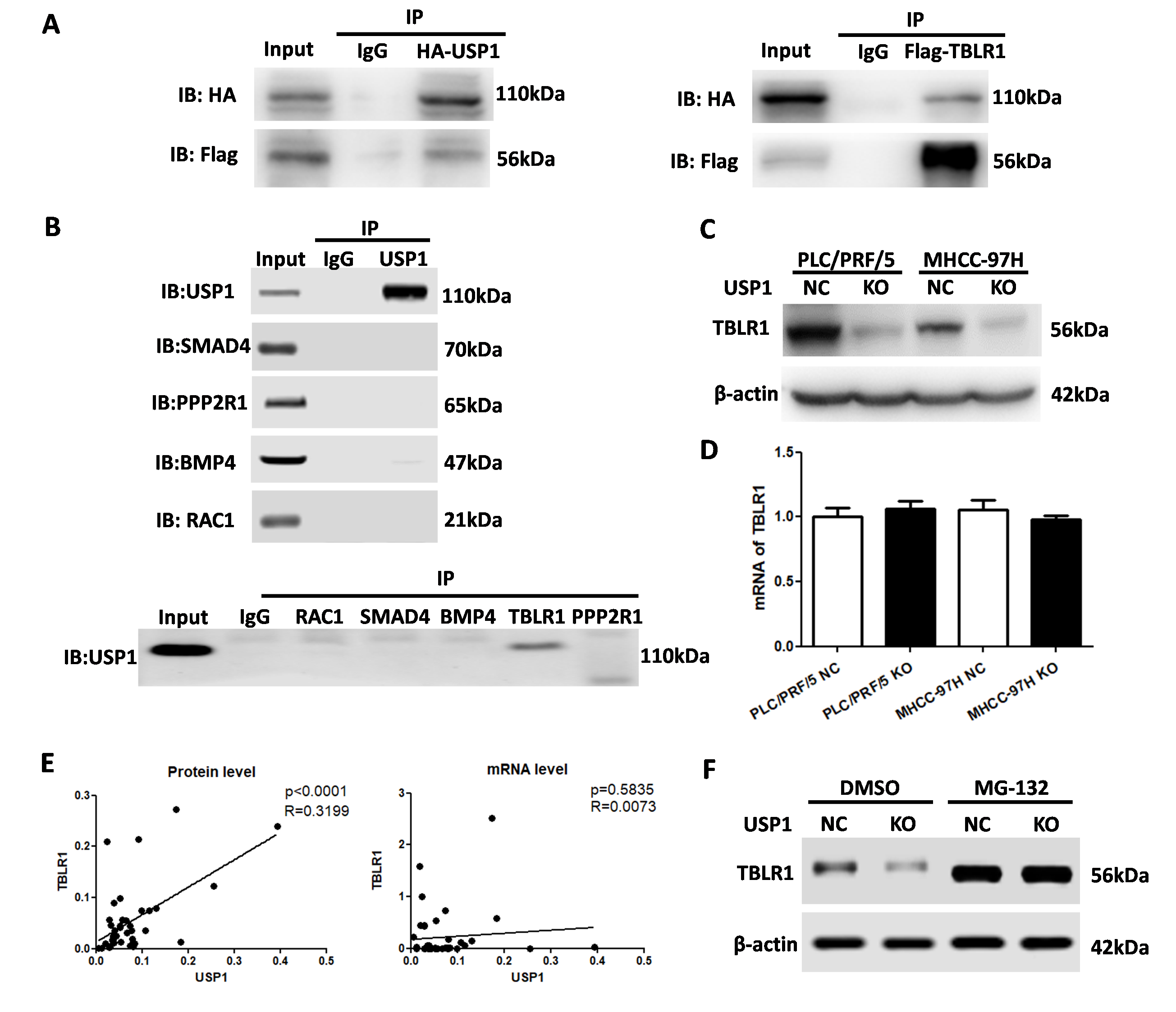

Supplement: FIGURE S4 — USP1 interacts with TBLR1 and correlates with TBLR1 protein level. (A) Co-IP assays of USP1-HA and TBLR1-Flag in 293T cells. (B) Co-IP assays of USP1-HA and RAC1, SMAD4, BMP4 or PPP2R1 in 293T cells. (C) TBLR1 expression in USP1-NC and USP1-KO cell lines. (D) TBLR1 mRNA level in USP1-NC and USP1-KO cell lines. (E) Correlation of USP1 and TBLR1 in patient samples. (F) TBLR1 is degraded in a proteasome-dependent manner and inhibited by MG-132. [file Image_4.TIF]

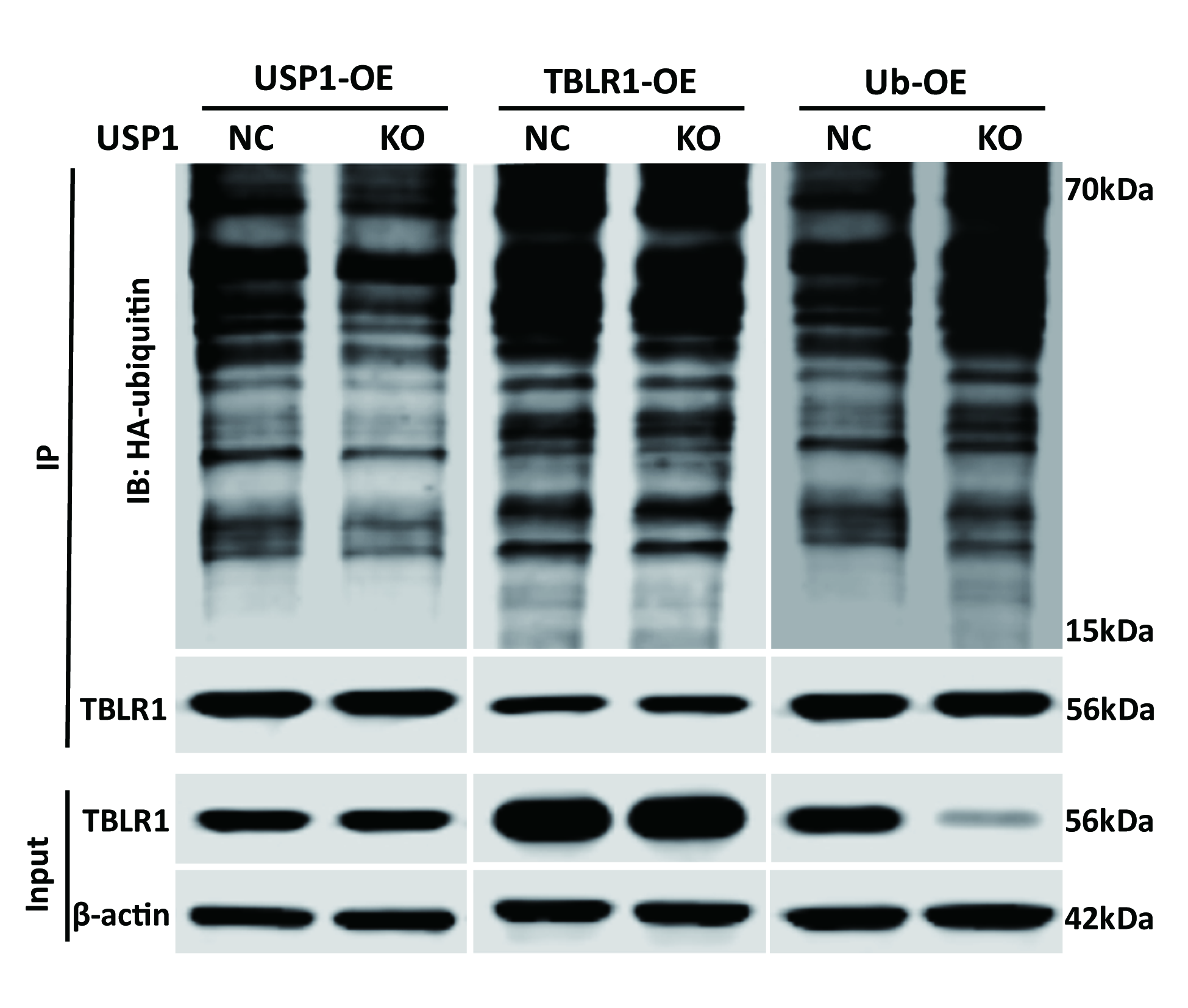

Supplement: FIGURE S5 — Ub assay in overexpression system by using USP1, TBLR1, and Ub plasmid. [file Image_5.TIF]
