# Supplementary material for: USP1 Maintains the Survival of Liver Circulating Tumor Cells by Deubiquitinating and Stabilizing TBLR1
Source: Front Oncol. 2020 Sep 25;10:554809. doi: 10.3389/fonc.2020.554809 (PMC7545832; doi:10.3389/fonc.2020.554809)
Supplement: TABLE S1 — Expression of USP1 and correlation with clinical characteristics of HCC patients (n = 217). [file Table_1.DOC]

Table S1. Expression of USP1 and correlation with clinical characteristics of HCC patients (n = 217)

|  | **USP1 expression** | |  |
| --- | --- | --- | --- |
|  | **Low (125)** | **High (92)** | **p-value** |
| **Gender** |  |  | 0.583 |
| Male | 101 | 77 |  |
| Female | 24 | 15 |  |
| **Age, years** |  |  | 0.506 |
| ≤50 | 49 | 32 |  |
| >50 | 76 | 60 |  |
| **Serum AFP, ng/mL** |  |  | 0.013 |
| ≤20 | 32 | 11 |  |
| >20 | 93 | 81 |  |
| **Tumor size, cm** |  |  | 0.696 |
| ≤5 | 88 | 67 |  |
| >5 | 37 | 25 |  |
| **Tumor number** |  |  | 0.028 |
| Single | 104 | 65 |  |
| Multiple | 21 | 27 |  |
| **Vascular invasion** |  |  | 0.383 |
| No | 74 | 49 |  |
| Yes | 51 | 43 |  |
| **Liver cirrhosis** |  |  | 0.495 |
| No | 20 | 18 |  |
| Yes | 105 | 74 |  |
| **HBsAg** |  |  | 0.42 |
| **-** | 18 | 17 |  |
| **+** | 107 | 75 |  |
| **Tumor differentiation** |  |  | 0.057 |
| I–II | 95 | 59 |  |
| III–IV | 30 | 33 |  |
| **Serum ALT, U/L** |  |  | 0.905 |
| ≤75 | 108 | 80 |  |
| >75 | 17 | 12 |  |

*Pearson’s chi-square test was used. P < 0.05 was considered as statistically significant
